# Audiovisual Simultaneity Judgment and Rapid Recalibration throughout the Lifespan

**DOI:** 10.1371/journal.pone.0161698

**Published:** 2016-08-23

**Authors:** Jean-Paul Noel, Matthew De Niear, Erik Van der Burg, Mark T. Wallace

**Affiliations:** 1 Neuroscience Graduate Program, Vanderbilt Brain Institute, Vanderbilt University Medical School, Vanderbilt University, Nashville, TN, 37235, United States of America; 2 Vanderbilt Brain Institute, Vanderbilt University Medical School, Vanderbilt University, Nashville, TN, 37235, United States of America; 3 Medical Scientist Training Program, Vanderbilt University Medical School, Vanderbilt University, Nashville, TN, 37235, United States of America; 4 Department of Experimental and Applied Psychology, Vrije Universiteit Amsterdam, Amsterdam, The Netherlands; 5 School of Psychology, University of Sydney, Sydney, Australia; 6 Department of Hearing and Speech Sciences, Vanderbilt University Medical Center, Nashville, TN, 37235, United States of America; 7 Department of Psychology, Vanderbilt University, Nashville, TN, 37235, United States of America; Harvard Medical School, UNITED STATES

## Abstract

Multisensory interactions are well established to convey an array of perceptual and behavioral benefits. One of the key features of multisensory interactions is the temporal structure of the stimuli combined. In an effort to better characterize how temporal factors influence multisensory interactions across the lifespan, we examined audiovisual simultaneity judgment and the degree of rapid recalibration to paired audiovisual stimuli (Flash-Beep and Speech) in a sample of 220 participants ranging from 7 to 86 years of age. Results demonstrate a surprisingly protracted developmental time-course for both audiovisual simultaneity judgment and rapid recalibration, with neither reaching maturity until well into adolescence. Interestingly, correlational analyses revealed that audiovisual simultaneity judgments (i.e., the size of the audiovisual temporal window of simultaneity) and rapid recalibration significantly co-varied as a function of age. Together, our results represent the most complete description of age-related changes in audiovisual simultaneity judgments to date, as well as being the first to describe changes in the degree of rapid recalibration as a function of age. We propose that the developmental time-course of rapid recalibration scaffolds the maturation of more durable audiovisual temporal representations.

## Introduction

It is well established that the integration of, or interaction between, different sensory modalities–usually conveying redundant information–results in dramatic perceptual and behavioral benefits [1, 2]. Examples of such multisensory-mediated benefits include enhanced detection and discrimination [3, 4] facilitated target localization [5–9] and speeded reaction times [10–13].

Early seminal papers in animal models delineating the governing principles dictating multisensory interactions showed that among other factors, a key component dictating multisensory integration is the temporal proximity between the stimuli to be combined [14, 15]. That is, the closer in time two multisensory events are to one another, the more likely that the pairing of these stimuli will result in enhanced neural activity (relative to the strongest of the unisensory responses) and behavioral gains (relative to best unisensory performance). This property (e.g., integration or interaction of sensory stimuli occurring close in time) is considered to be emergent from the fact that, ecologically, information from multiple sensory modalities originating from a single source (as opposed to distinct objects or events) are most likely to co-occur in time [2, 15].

At the neural level, the temporal tuning functions of individual multisensory neurons–that is, their firing rate profile as a function of stimuli onset asynchrony (SOA)—peak near true synchrony, yet show multisensory gain (i.e., enhanced neural response in comparison to unisensory stimulation) over temporal windows extending for hundreds of milliseconds [14]. The fact that this integration takes place over a temporal “window,” rather than as a point function, is likely the result of the fact that neural representations need to accommodate different propagation times and neural transduction speeds of energies in the different senses [12, 16–18].

From a developmental perspective, it has been shown that multisensory neurons and their associated integrative properties mature over a protracted period of development [19–23], allowing for an experience-dependent shaping of neural representations to match the spatiotemporal features of the environment. Indeed, studies have shown remarkable plasticity in the development of these processes, such that changes in the spatiotemporal structure of the early sensory world results in the development of integrative properties that match these statistics [24, 25]. The animal neurophysiological study of multisensory processes in aging, as opposed to development, has been much less explored.

Complementary behavioral studies in humans have reinforced the fact that multisensory benefits are observed over relatively large audiovisual temporal asynchronies, and have led to the concept of a “temporal window of simultaneity” (TWS), within which audiovisual stimuli are integrated and perceptually bound [26–29]. Rather than being a fixed construct, the size of multisensory TWS has been shown to be highly plastic [30]. Indeed, the ability to discern temporal structure in paired audiovisual stimuli emerges very early in human development. Lewkowicz (1996) [31] has shown that infants as young as 2-months old can detect multisensory asynchronies, and it is well established that there are profound changes in the evaluation of audiovisual temporal relations as development progresses [32–34]. Furthermore, it has been recently shown that these changes continue well into adolescence [35–36]. Interestingly, multisensory processing may not only be highly plastic early in development, but also later in life. Emerging evidence has indicated that the width of the TWS tends to increase in size with age, as evaluated using both temporal order [37–40] and simultaneity judgments [41–43]. (Although see [44] for discrepant findings with regard to simultaneity judgments). While it appears likely that changes in the size of the TWS during development is related to the maturation of the sensory systems themselves, adjustment in the size of these windows in older age may more closely reflect the need for greater accumulation of evidence in light of the deteriorating sensory periphery. Thus, variations in the size of the TWS in development and aging may not reflect the same underlying mechanistic process(es).

In addition to being modified as a function of age, audiovisual temporal function also appears to be plastic and based on the history of an individual’s sensory experience. Thus, upon extensive exposure to asynchronous audiovisual stimuli, the point of subjective simultaneity (PSS–the stimulus onset asynchrony at which paired audiovisual stimuli are perceived as simultaneous) shifts in the direction of the repeatedly presented asynchrony [45, 46]. Recent findings have extended this work to show that temporal recalibration can also occur on a much more rapid timescale, being driven on the basis of the temporal structure of the previous trial alone. In other words, Van der Burg, Alais and Cass (2013) [47] found clear evidence that when participants performed a simultaneity judgment task, the PSS was contingent upon the modality order (i.e., visual first, auditory first) of the preceding trial. This rapid recalibration effect can be observed using simple audiovisual stimuli such as a flash in combination with a beep [47–49], but also for perceptually complex stimuli such as audiovisual speech [50].

Taken together, the literature suggests that audiovisual simultaneity judgment is highly plastic and changes both during development and adulthood, though importantly there is no report spanning the entire lifespan. These aging effects appear to be adaptive in that they allow for incorporating sensory statistics–which arguably change with age and (mal)function of peripheral sensory organs–into one’s representation of the world. Equally adaptive are rapid temporal recalibration effects, which allow for updating sensory expectations on a moment-to-moment basis. Indeed, recent reports suggest that certain psychiatric disorders, such as Autism Spectrum Disorder (ASD; [51, 52]), exhibit a deficit in rapid recalibration to audiovisual stimuli. However, these studies may be confounded by the fact that ASD populations tested are generally of younger age (i.e., children), and there is no report regarding the development of rapid audiovisual recalibration as a function of age. Lastly, the joint analysis of audiovisual simultaneity judgment, and the degree to which audiovisual representations rapidly recalibrate as a function of age may shed new light with regard the developmental hierarchy between the two. It is conceivable that stable and cumulative temporal filters (i.e., TWS) are strongly impacted by the moment-to-moment changes in these representations i.e., rapid recalibration [53].

In the present study, we were interested in exploring changes in audiovisual simultaneity judgment (i.e., the TWS), the ability to recalibrate to multisensory asynchrony, and the relationship between them across the lifespan. To do this, participants (ranging in age from 7 to 86 years) performed a simultaneity judgment task in which they reported whether the audiovisual stimulus were synchronous or asynchronous. The audiovisual stimuli were either simple (a flash in combination with a beep) or complex (audiovisual speech). Our overarching hypothesis was that both the size of the TWS as well as the magnitude of rapid recalibration would follow a U-shaped pattern across lifespan. Early in life, sensory filters are arguably still shaping (adapting to sensory statistics), while later in life these filters may broaden again as to allow for further accumulation of evidence, as sensory skills decrease. Further, we conjecture that changes in rapid recalibration may occur earlier in development as compared to overall changes in the size of the TWS, as rapid recalibration may represent a measure of variability that ultimately impacts more stable temporal representations such as the TWS. That is, we hypothesize that changes in trial-to-trial variability in the judgment of simultaneity, as a function of the nature of the previous trial (audio-lead vs. visual-lead), may ultimately lead to more durable changes in the size the TWS. Thus, we postulate that developmental changes in rapid recalibration may precede alterations of TWS size. Lastly, we predict that changes in rapid recalibration and TWS will be evident earlier in development for low-level stimuli, which may subsequently scaffold the maturation of higher-order audiovisual representations (e.g., speech).

## Methods

### Participants

**Fig 1:**
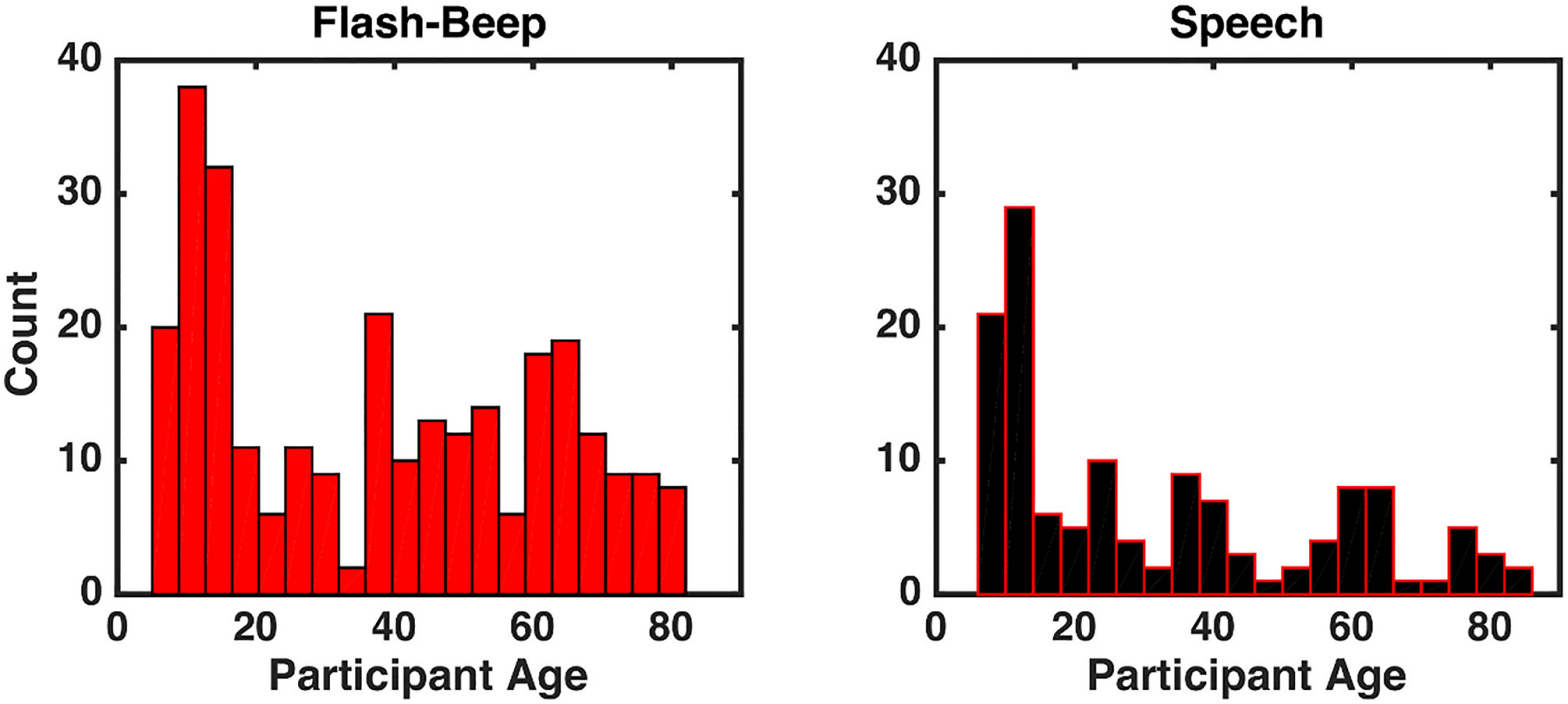
Histogram of participants’ age as a function of stimulus type. A) Distribution of the ages of participants who were presented with Flash-Beep stimuli. B) Distribution of the ages of participants who were presented with Speech stimuli.

220 participants took part in this study (142 females; age range = 7–86 years old), 156 were submitted to Flash-Beep stimuli, while the remaining 64 were presented with audiovisual Speech stimuli (see Table 1 for age breakdown). See Fig 1 for a histogram of the distribution of ages as a function of stimuli type. All participants had correct or corrected-to-normal visual acuity, self-reported normal hearing, and were naïve as to the purpose of the experiment. None of the participants had a history of either psychiatric or neurological condition. Written informed consent was obtained from all participants, and Vanderbilt University Medical Center’s Institutional Review Board approved the study. In the case of minors, caretakers or guardians on behalf of the minors/children enrolled in the study provided written informed consent.

**Table 1:** Number of participants as a function of age and stimuli presented. Number of participants within given age (left column) bins for both the Flash-Beep stimuli (middle column) and Speech stimuli (right column).

	Flash-Beep Stimuli	Speech Stimuli
7–10	9	3
11–15	27	18
16–20	11	3
21–25	4	4
26–30	5	2
31–35	2	1
36–40	9	6
41–45	6	2
46–50	13	1
51–55	12	5
56–60	10	1
60–65	17	6
66–70	12	2
71–75	7	5
76–80	9	4
80+	3	1
**Total**	156	64

### Materials and apparatus

Two distinct categories of audiovisual stimuli were presented: Flash-Beep (simple stimuli) or single syllable utterances (complex stimuli). In terms of the Flash-Beep stimuli, the visual component consisted of a white ring circumscribing a fixation cross on a black background, and was 17.3° of visual angle. Visual stimulus duration was 10 ms (presented on a Samsung Sync Master 2233RZ monitor at 100 HZ). The auditory stimuli consisted of a 3500 Hz pure tone with a duration of 13 ms. With regard to the speech stimuli, syllable utterances were displayed. This stimuli consisted of two audiovisual clips of a female speaker uttering single instances of the syllables /ba/ and /ga/. Visual stimuli were, down-sampled to a resolution of 400 × 400 pixels spanning 17.3° of visual angle, converted from color to grayscale, and cropped to a square. Presentations were shortened to 2 s, and each presentation included the entire articulation of the syllable, including pre-articulatory gestures (for similar stimuli see [54], as well as [55]). Visual stimuli were presented at a distance of approximately 60 cm from the participants and auditory stimuli were presented binaurally via headphones. All stimuli were presented using MATLAB (MathWorks Inc., Natick, MA) software with the Psychophysics Toolbox extension [56, 57] with their duration and temporal onsets confirmed via a Hameg 507 oscilloscope.

### Procedure

Participants sat inside a light- and sound-attenuating WhisperRoom^™^ (Model SE 2000; Whisper Room Inc), and were instructed to judge whether the audiovisual events were synchronized or not (i.e., a classical simultaneity judgment task). Participants were asked to fixate toward a fixation cross at all times and instructions emphasized accuracy only. A closed circuit infrared camera monitored their compliance with the task throughout the experiment. Both in the case of the Flash-Beep and the Speech stimuli each trial was composed of a 501–1500 ms fixation (uniform random) period, a stimulus presentation interval (note that the duration of this interval varied dependent upon stimulus type [Flash-Beep versus Speech] and as specified in the *Materials and Apparatus* section), a 250 ms fixation period, and a response screen. Following a response via button press, the subsequent trial began with the 501–1500 ms fixation. In the case of the Flash-Beep stimuli, participants were presented with SOA of 0, ±10, ±20, ±50, ±80, ±100, ±150, ±200, ±250, and ±300 ms (we denote audio-leading stimuli by negative SOAs). Twenty repetitions were presented for each SOA condition resulting in a total of 380 **b** are typically wider than those reported for “flash-beep” stimuli. Consequently, the presentation of different SOAs for these two types of stimuli allows for a more accurate estimate of the TWS. No practice trials were administered before initiation of the experiment.

### Analysis

Reports of synchrony as a function of SOA were compiled for each participant and stimulus type both independently and dependently of the nature of the precedent trial (i.e., the conditional that t-1 was either an audio- or visual-leading presentation). In the case of the latter (binning on the conditional of the nature of the precedent trial), trials preceded by synchronous audiovisual events (SOA = 0 ms) were discarded from further analyses. These reports of synchrony were then fitted (via non-linear squares method) with a Gaussian distribution whose amplitude, mean, and standard deviation (SD) were free parameters (see Eq 1). The amplitude was free to vary between 0 and 1. The mean of the Gaussian was taken as the point of subjective simultaneity (PSS), and the SD was taken as a measure of the TWS [47–49, 52]. The shape of the normal distribution proved to accurately describe the reports of synchrony (mean R^2^ = 0.911), and we were not able to find a significant difference in the Goodness-of-Fit across different ages (independent samples over sliding bins of 11 participants–see below–all *p* values > 0.12). Similarly, the mean amplitude of such distributions–putatively indicative of response biases–did not differ as a function of age (all *ps* > 0.24).

In order to index rapid recalibration effects, the PSS shift (ΔPSS = PSS audio leading on t-1 –PSS visual leading on t-1) as well as the TWS shift (ΔTWS = TWS audio leading on t-1 –TWS visual leading on t-1) were computed.

$$P\left(response|SOA\right)=\;amp\;\times \;exp^{-\left(\frac{{\left(SOA-PSS\right)}^{2}}{2SD^{2}}\right)}$$ (1)

In order to avoid computing conjugate central tendency measures (i.e., mean) that can disregard the main independent measure of interest here (i.e., age), we adopted a sliding window approach. Dependent variables (PSS, TWS, ΔPSS, and ΔTWS) were sorted according to participant’s age, and then a sliding window of width 11-participants was moved on a subject-per-subject basis. Hence, no pre-defined binning of participants as a function of age was undertaken. At each position of the window we computed the average PSS, TWS, ΔPSS, and ΔTWS. These measures are utilized in order to determine *i*) effects across ages and stimuli type, *ii*) within conditions, the ages at which dependent variables are significantly different from that of the youngest age, and *iii*) significantly different from the minimum value for the particular dependent variable. The first analysis allow for determining the age at which certain processes reach maturity and whether this age is different across stimulus type, while the latter analysis is undertaken in order to explore the shape of the different developmental time-courses (i.e., to determine whether the dependent variables exhibit similarities between childhood and old age). Inferential statistics throughout consisted of independent t-tests, and to correct for multiple comparisons we conduct non-parametric false discovery rate (FDR p < 0.05; [58]) on the resulting p-values.

## Results

### The temporal window of simultaneity exhibits a protracted developmental time-course

**Fig 2:**
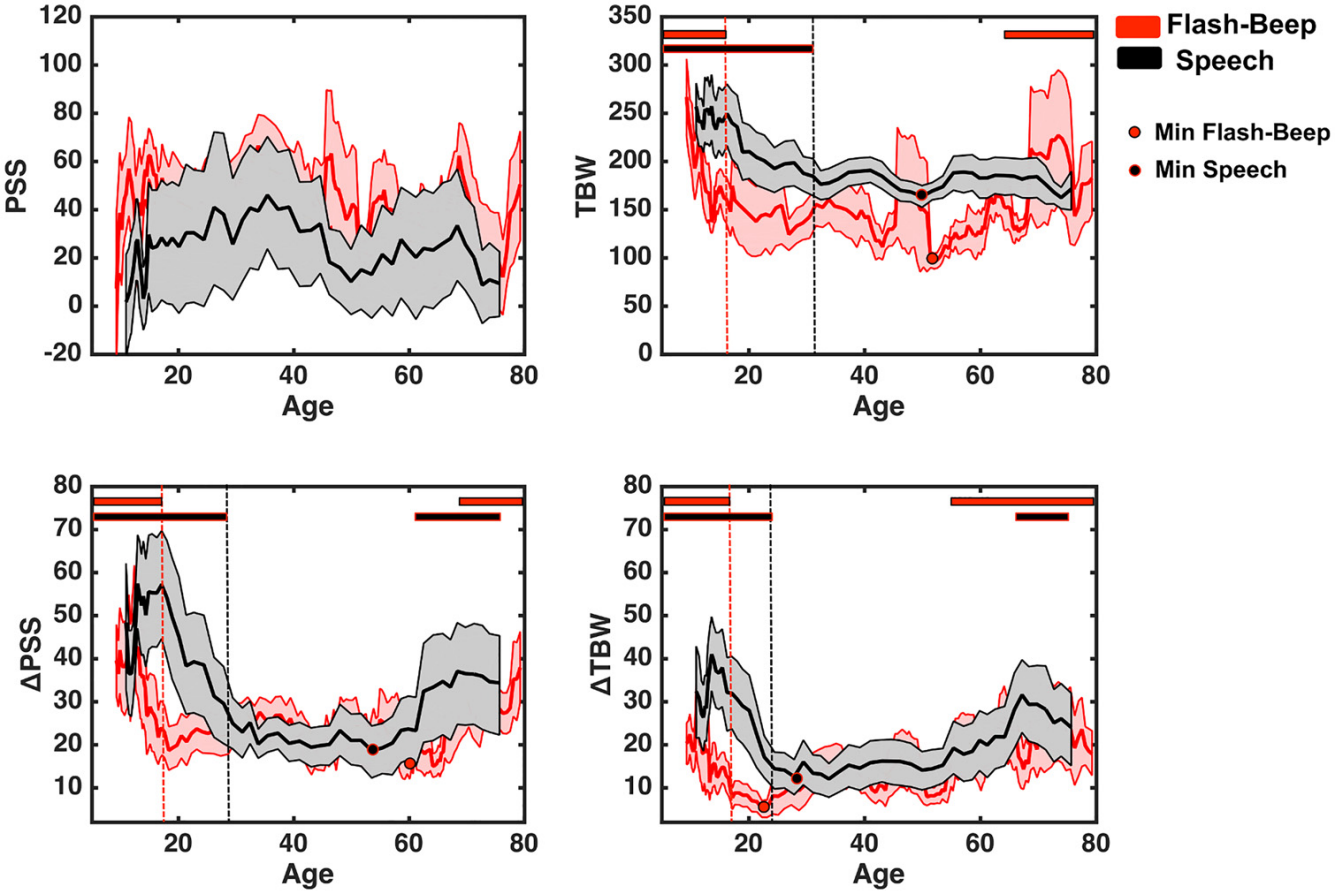
Simultaneity judgment and rapid recalibration as a function of stimuli complexity and age. Although no change is apparent in PSS (upper left), temporal window of simultaneities for both Flash-Beep (red) and Speech (black) stimuli portray protracted developmental time-courses (upper right). Similarly, rapid recalibration effects, both in terms of change in PSS (lower left) and TWS (lower right) as a function of the nature of the immediately precedent trial follow protracted developmental time-course, and a U-shape, indexing greater tendency to rely on recent perceptual experience later in life. Solid lines represent the mean of the 11-participant wide window centered at the particular age, shaded areas around the solid lines represent +/- 1 S.E.M. Dashed vertical lines represent the first age-point at which within condition values differed from the first time-point (thus, age at which the particular perceptual process reached maturity). The colored circles represent the minimum value for either the raw TWS or the change in PSS or TWS as a function of age. And finally the solid horizontal lines at the top of the panels indicate significant differences (p < 0.05) from the minimal value (circle). Hence, if these solid horizontal lines are present both at earlier and later ages than the respective minimum, we categorize the particular time-course as being U-shaped.

We first characterized the TWS across lifespan for simple flash-beep stimuli. As illustrated in Fig 2A (upper right panel), results demonstrated that the TWS was largest for the youngest participants (mean age = 9.62 years, TWS = 222.45 ms) and narrowed progressively until becoming significantly different from this initial value at about age 17 (mean age = 17.34 years, TWS = 163.46, p < .05). The smallest TWS was seen slightly after 50 years of age (mean age = 51.35 years, TWS = 96.88 ms), where the TWS was significantly different from both the youngest participants (17 and younger, as specified above) and from those participants older than 64 years of age (all ps < 0.05, whom exhibited a larger TWS). Thus, the TWS increased again after 50 years of age, with significant differences emerging at age 64. Consequently, the size of the TWS for flash-beep stimuli exhibited a U-shape pattern as a function of age.

The TWS for speech stimuli displayed a similar developmental pattern to that seen for simple stimuli, yet its developmental time-course was considerably delayed. Results demonstrated that the TWS for speech stimuli was largest for the younger participants (mean age = 11.67 years, TWS = 248.56 ms) and diminished in size becoming significantly different from this value at about age 31 (mean age = 31.67 years, TWS = 188.46, p < 0.05). Following the age of 31 years old, no further changes were apparent in the size of TWSs. The smallest TWS for Speech stimuli were displayed when the sliding window was centered at age 49 (TWS = 162.12), yet this group was not statistically significant from any other age groups after age 29. Thus, in contrast to the pattern seen for simple stimuli, the TWS for speech stimuli did not appear to follow a U-shaped pattern.

In striking contrast to these dynamic, developmentally mediated changes in the TWS across lifespan, the raw value of the PSS did not appear to significantly change across development for neither simple nor complex stimuli (all ps > 0.91).

### The magnitude for rapid recalibration changes throughout the lifespan

As illustrated in Fig 2, the change in PSS (lower left) and TWS (lower right) as a result of the nature of the immediately preceding trial (audio- vs. visual-leading) was highly dependent on age. In regard to rapid recalibration changes in the PSS (ΔPSS), both for flash-beep and speech stimuli, young children exhibited a large inter-trial effect (peak effects at age 12.10 years for ΔPPS [50.52 ms] for flash-beep stimuli and peak effects at age 16.35 years for ΔPPS [57.31 ms] for speech stimuli). The magnitude of these temporal recalibration effects decreased with development. Thus, effects were significantly different from their initial value at age 18 for the flash-beep stimuli (ΔPPS = 23.56 ms, p < 0.05) and at age 29 for the speech stimuli (ΔPPS = 25.61 ms, p < 0.05). The smallest magnitude of rapid recalibration for flash-beep stimuli was observed at about age 60 (mean age = 60.03, ΔPPS = 15.04 ms). This minimum, in addition to being significantly different from those under age 18, was also statistically different from the ΔPPS displayed by subjects older than 67 years old (ΔPPS = 22.67 ms, p < 0.05). In a similar fashion, the smallest amount of rapid recalibration for speech stimuli was present when the sliding window was centered on age 53 (mean age = 53.45, ΔPPS = 18.63 ms). This value was significantly different from that seen in those age 61 and older (ΔPPS = 32.53 ms, p < 0.05), and from those age 29 and younger (as aforementioned). Thus, across lifespan, changes in the ability for rapid recalibration appear to follow a U-shaped pattern.

With regard to changes in the TWS (Fig 2, lower right panel) a similar change in rapid recalibration was observed. Specifically, the ΔTWS was largest at the youngest tested ages and progressively diminished, being significantly smaller at age 18 (ΔTWS_18_ = 9.83 ms vs. ΔTWS_initial_ = 21.24 ms) for flash-beep stimuli and at age 22 (ΔTWS_22_ = 16.20 ms vs. ΔTWS_initial_ = 33.07 ms) for speech stimuli. The minimum values for the ΔTWS was seen in individuals’ aged approximately 22 and 28 years old (ΔTWS = 5.20 ms, ΔTWS = 12.06 ms) for flash-beep and speech stimuli, respectively. These minima proved to be significantly smaller than the ΔTWS displayed by older participants, with significant differences emerging at age 56 for flash-beep stimuli (ΔTWS = 19.18 ms, p < 0.05) and at age 64 for speech stimuli (ΔTWS = 27.33 ms, p < 0.05). Thus, much like as for the ΔPSS, the ΔTWS displayed a U-shape as a function of age.

### Rapid recalibration and simultaneity judgment are correlated as a function of age

**Fig 3:**
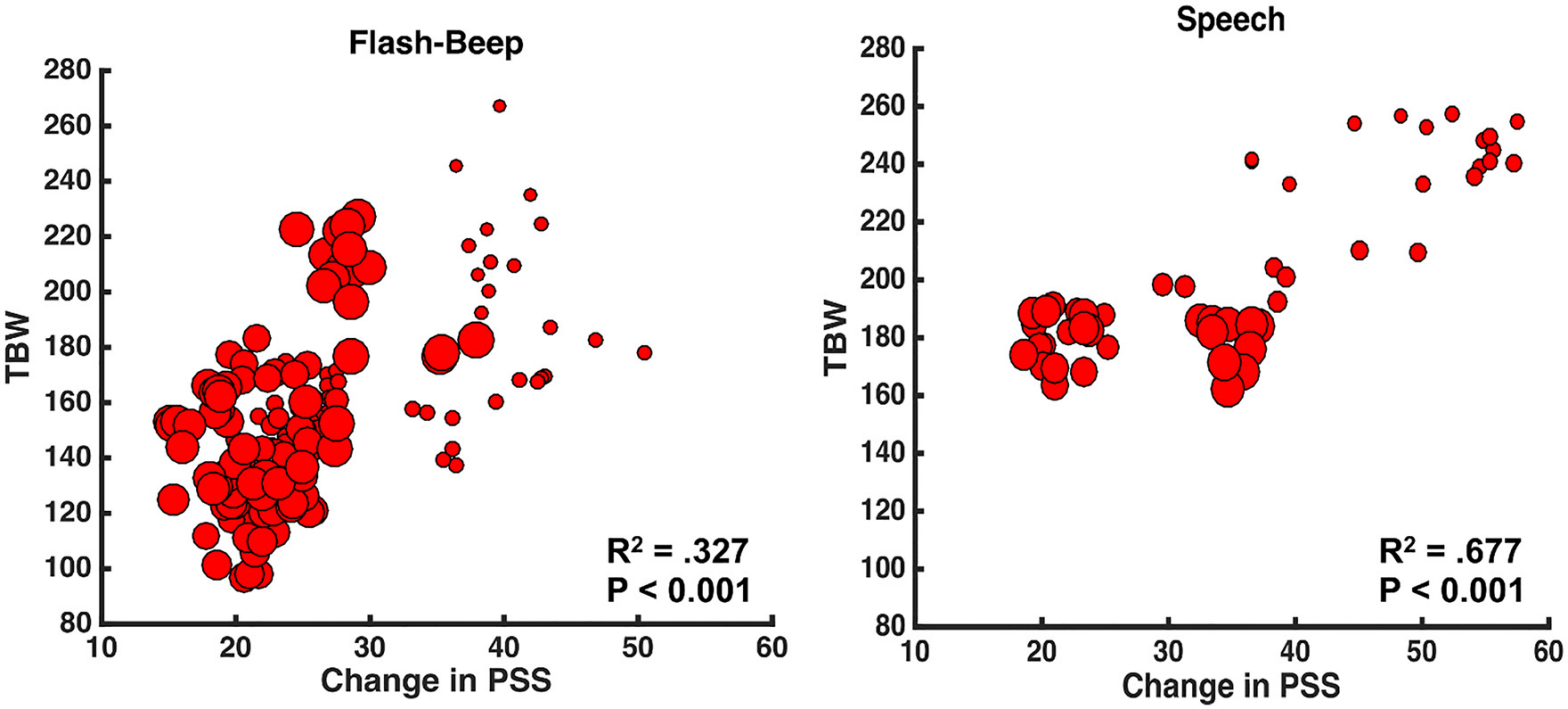
Correlation between the size of participant’s TWS and rapid recalibration (ΔPPS). Left panel demonstrated a significant correlation between TWS and ΔPPS for Flash-Beep stimuli, while right panel demonstrates the same relation for Speech stimuli. The size of the dots indexes age of the participant represented, conveying the fact that these correlations seem to be largely driven by age.

In order to attempt to further relate the degree to which the nature of the immediately preceding multisensory trial (audio- vs. visual-leading) impacts ongoing simultaneity judgment, and how this relationship changes throughout the lifespan, we conducted additional correlational analyses. Van der Burg et al., (2013) [47] showed a positive correlation between the size of an individual’s TWS and their magnitude of rapid recalibration (mean age in [47], was 26.6 years old). Here we find a similar relationship when using a large sample size, both for the flash-beep (R^2^ = 0.327, p < 0.001, Fig 3. left panel) and speech (R^2^ = 0.677, p < 0.001, Fig 3. right panel) stimuli. Importantly, however, and as illustrated in Fig 3 (in which larger dots represent older ages), this correlation seems to be largely driven by age. That is, early in life, TWS are large (i.e., temporal acuity is poor) and individuals appear to demonstrate a greater degree of rapid recalibration. Indeed, when accounting for age (via partial correlations), the relationship between TWS size and ΔPSS failed to survive for both flash-beep and speech stimuli (both p > 0.29).

## Discussion

In the current study we provide the most comprehensive description to date of the relationship between audiovisual simultaneity judgment (i.e., the TWS) and the magnitude for rapid temporal recalibration (ΔPSS) across lifespan. Our results demonstrate that changes in the TWS and ΔPSS: i) take place over a protracted developmental time course, ii) appear to mature earlier for simple (i.e., flash-beep) stimuli when compared with more complex (i.e., speech) stimuli, and iii) followed a U-shaped pattern across lifespan (being smallest at intermediate ages).

Distinct from prior work in this realm, we did not bin participants within pre-defined age groups, but adopt a more continuous measure by employing a sliding-window approach. Similar to prior accounts using more discreet age distributions, the current findings indicate that the development of the TWS follows a protracted time-course, reaching maturity at around 17 years of age for simple stimuli and, surprisingly, not until about 31 years of age for speech stimuli. Overall our findings are consistent with prior studies demonstrating that TWSs become narrower with age [35, 36]. Specifically, [35] estimated that the TWS for flash-beep stimuli reached maturity a few years later than the current estimates (i.e., between 18 and 23). This minor discrepancy is likely due to the binning of participants by age (in [35]), not allowing for the more continuous and fine grain estimate that was accomplished here. Further, it must be highlighted that in the current project we define ‘maturity’ as not being statistically different from the ‘most mature’ value observed within the dataset. That is, we pick the extreme values (e.g., smallest TWS), and compare the rest of the ages to this extreme value. This approach may engender an overly stringent definition of sensory ‘maturity’.

In terms of the narrowing of the TWS for speech stimuli, Lewkowicz and Flom (2014) [59] recently showed that these windows are still larger at age 6 when compared with adults. However, to the best of our knowledge, a more comprehensive analysis spanning from development throughout old age, as is reported here, has not been previously described. Recently, our group [60] has demonstrated that although older subjects experience the McGurk illusion [61] more frequently than younger participants, the dependency of the illusion on the temporal structure of the combined stimuli did not exhibit a developmental time-course. Thus, although the current study and that of Hillock-Dunn et al. [35] differ in a number of methodological aspects, collectively they both show that despite broader temporal profiles for audiovisual simultaneity judgments of speech-related stimuli, they are relatively adult-like in their temporal profile for fusing discordant auditory and visual tokens into a novel percept (i.e., perceiving the McGurk illusion). An interesting question for future studies thus lies in identifying both the sensory and cognitive compensatory mechanisms young participants may employ in order to correctly identify syllables using representations that have relatively poor audiovisual temporal resolution. Analogously, it will be interesting to compare within subjects and across development the time-course exhibited by audiovisual speech simultaneity judgments (as done here) and binding (via measures such as the McGurk illusion, as in [60]).

Indeed, the exact relationship between the degree to which an individual rapidly recalibrates as a function of immediately prior sensory information and their temporal window of simultaneity is likely a complex one, depending on both bottom-up and top-down factors, and differing according to the nature of the stimuli presented. That is, semantic relationships appear to influence the detection of synchrony for speech stimuli as the TWS has been reported to be wider for congruent audiovisual speech pairs (e.g. visual /da/ paired with auditory /da/) than for incongruent speech pairs (e.g. visual /ga/ paired with auditory /ba/; [17]). Similarly, the TWS is wider for speech when the gender of the auditory and visual streams matches [62]. Interestingly, the capacity for semantic relationships to influence temporal acuity may only apply, or apply more strongly, to speech stimuli, as temporal precision is equivalent for mismatched and matched dynamic, non-speech stimuli [63]. On the other hand, rapid recalibration effects have been demonstrated for both low-level [47] and higher-level speech [50] stimuli. These latter effects, however, appear to be independent of the identity of the prior speaker as well as if the prior speech cues. Therefore, it appears that semantic representations may not only affect the size of the TWS, but also the relationship between this window and the degree to which individuals rapidly recalibrate.

Finally, when considering the older ages in our study population, we saw that the TWS increases again in size for aging populations for flash-beep stimuli [41–43], suggesting that the lifespan time-course for audiovisual simultaneity judgment follows a U-shaped pattern, at least for the binding of simple stimuli. In contrast, an increase in the TWS for speech stimuli in older populations was not apparent, suggesting that additional factors (e.g., lip-reading) may play an important role in keeping these ecologically relevant windows narrow in late-adulthood, and thus allow for multisensory gains [64].

With regard to the manner in which individuals rapidly recalibrate to audiovisual asynchronies (as driven by the temporal structure of the preceding trial), results demonstrated that both for simple and complex stimuli, this adaptation follows a protracted developmental time-course (maturity at about 18 years of age for flash-beep stimuli, and at age 29 for speech stimuli). Further, in older individuals, the degree to which immediately precedent perceptual experiences impact current simultaneity judgment appears to increase again, being significantly different from younger adults at age 67 and 61 for flash-beep and speech stimuli, respectively. Thus, for both simple and complex stimuli rapid recalibration throughout the lifespan appears to follow a U-shape. To the best of our knowledge this constitutes the first report of rapid recalibration in development as well as in aging. Prior work has reported reduced (slow) adaptation to repeatedly presented audiovisual asynchronies in elder populations [41, 42]. We do not find these two findings contradictory, as recent reports have highlighted the fact that immediate and prolonged recalibration effects are independent from one another and may follow distinct time-courses [65, 66].

Lastly, the current data supports the notion that the size of one’s TWS and the degree to which one will rapidly recalibrate as a consequence of recent perceptual experience are intrinsically linked. This is apparent in the fact that the width of TWSs and the change in PSS were positively correlated (see [47, 49]), although the correlation appears to be heavily influenced by age. Longitudinal studies, as opposed to the cross-sectional approach taken in the current study will be fundamental in a further effort to establish a causal link between the developmental time-course of rapid recalibration and multisensory temporal synchrony perception.

In addition to the fact that the present study was cross-sectional and not longitudinal in nature, and thus it is impossible to draw within-subjects conclusions, a few additional limitations must be acknowledged. First, a Gaussian fitting procedure was utilized as it is standard within the study of rapid recalibration (e.g., [47]); thus allowing for cross-study comparisons. Further, it proved to accurately describe the shape of the reports of synchrony (mean R^2^ = 0.911). However, the Gaussian fitting employed was by weighted by a least-squares method (as opposed to maximum likelihood), which although unlikely to change the reported results is formally improper as the raw data recorded were binomial (possible answers were either ‘synchronous’ or ‘asynchronous’). Lastly, some of the discrepancies relative to prior published work (i.e., [35]) with regard the reported age at which multisensory temporal processes reach maturity (e.g., TWS size; 17 years old here vs. 18–23 in [35]) may be due to the intrinsic statistical idiosyncrasies (e.g., auto-correlation, or propensity toward Type 1 error) between conducting analysis between discrete groups (as in [35]) versus a time-series approach (as conducted here).

Speculatively, these findings appear to suggest that rapid temporal recalibration effects may be intrinsically linked to the construction of more stable long-term audiovisual temporal constructs such as simultaneity judgment (i.e., the TWS). In such a context, rapid recalibration can be conceived of as a measure of variance, while the width of TWS can be taken as an index of central tendency. In this scenario, rapid recalibration effects are a result of dynamic moment-to-moment changes in audiovisual temporal filters and the associated neural representations; changes driven by the immediate statistical features of the external world. The accrual of experience in this dynamic context then may build a more stable longer-term representation (i.e., the TWS), which is likely a product of the architecture of the individual’s sensory processing apparatus (e.g., processing latencies and delays) as well as their prior weighted history of experience with the sensory world. Such a structure provides both the necessary adaptive flexibility to accommodate to immediate changes in the world as well as an important stability that takes into account individual differences in neural organization and experience.
